# Supplementary material for: Splice-Junction-Based Mapping of Alternative Isoforms in the Human Proteome
Source: Cell Rep. Author manuscript; Available in PMC 2020 Jan 15. (PMC6961840; doi:10.1016/j.celrep.2019.11.026)
Supplement: 3 [file NIHMS1546469-supplement-3.zip › DF2/PXD0010154/Esophagus-155-P24821-ASTEAEPEVDNLLVSDATPDGFR.pdf]

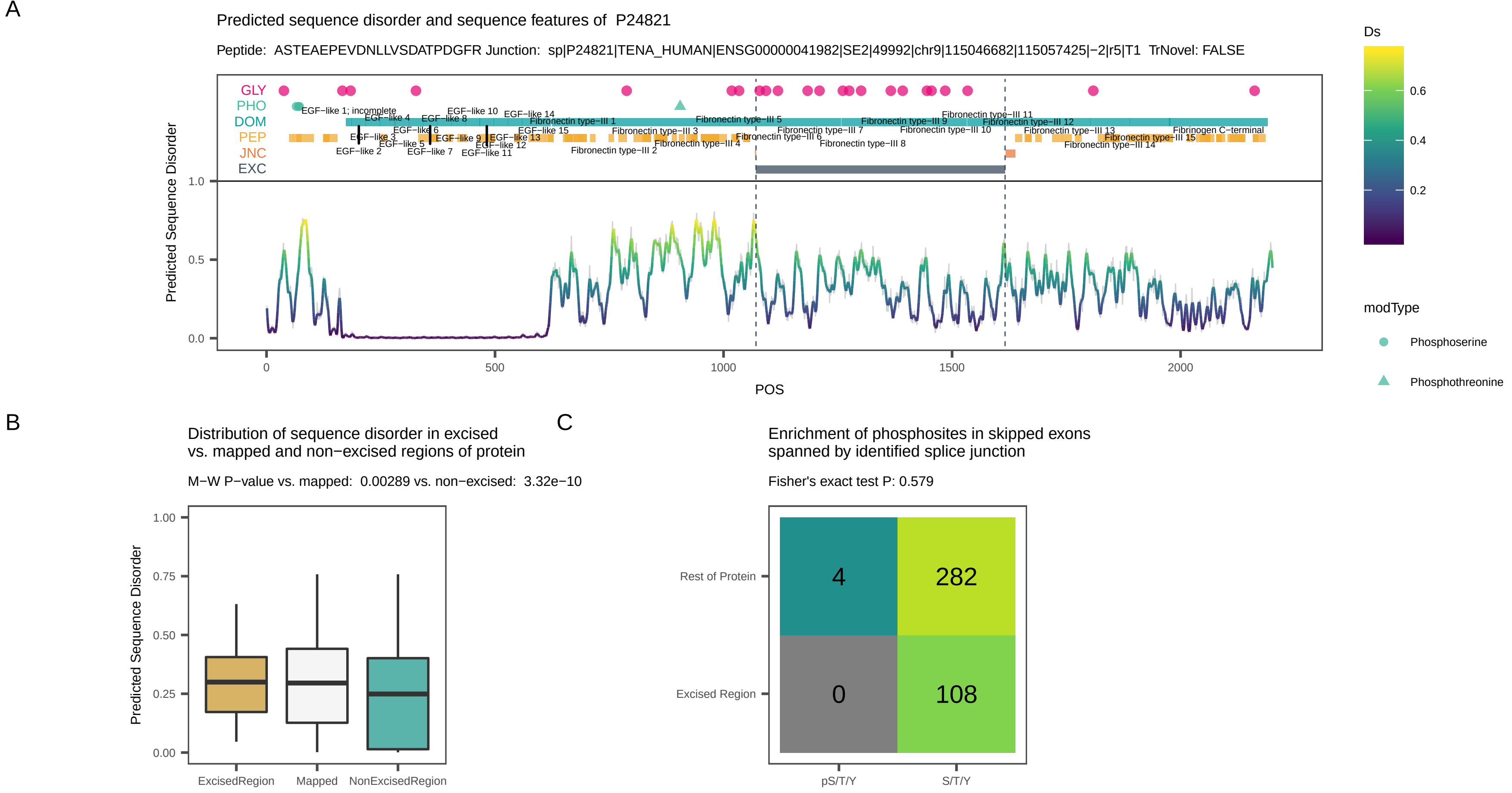
A
Predicted sequence disorder and sequence features of P24821
Peptide: ASTEAEPEVDNLLVSDATPDGFR Junction: sp|P24821|TENA_HUMAN|ENSG00000041982|SE2|49992|chr9|115046682|115057425|-2|r5|T1 TrNovel: FALSE
GLY
PHO
DOM
PEP
JNC
EXC
EGF-like 1; incomplete
EGF-like 2
EGF-like 3
EGF-like 4
EGF-like 5
EGF-like 6
EGF-like 7
EGF-like 8
EGF-like 9
EGF-like 10
EGF-like 11
EGF-like 12
EGF-like 13
EGF-like 14
EGF-like 15
Fibronectin type-III 1
Fibronectin type-III 2
Fibronectin type-III 3
Fibronectin type-III 4
Fibronectin type-III 5
Fibronectin type-III 6
Fibronectin type-III 7
Fibronectin type-III 8
Fibronectin type-III 9
Fibronectin type-III 10
Fibronectin type-III 11
Fibronectin type-III 12
Fibronectin type-III 13
Fibronectin type-III 14
Fibronectin type-III 15
Fibrinogen C-terminal
Predicted Sequence Disorder
1.0
0.5
0.0
0
500
1000
1500
2000
POS
Ds
0.6
0.4
0.2
modType
Phosphoserine
Phosphothreonine
B
Distribution of sequence disorder in excised vs. mapped and non-excised regions of protein
M-W P-value vs. mapped: 0.00289 vs. non-excised: 3.32e-10
Predicted Sequence Disorder
1.00
0.75
0.50
0.25
0.00
ExcisedRegion
Mapped
NonExcisedRegion
C
Enrichment of phosphosites in skipped exons spanned by identified splice junction
Fisher's exact test P: 0.579
Rest of Protein
Excised Region
4
282
0
108
pS/T/Y
S/T/Y
